# Blood, Brain, and Bypass: Moyamoya Syndrome Unmasked During Hematologic Optimization in an Adult With Sickle Cell Disease

**DOI:** 10.7759/cureus.106813

**Published:** 2026-04-10

**Authors:** Kian Memari, Emily Reinoso, Sergio A Rodriguez, Nicole P Alvarez, Lissette P Lazo, Shane Williams, Peter Cohen

**Affiliations:** 1 Family Medicine, Palmetto General Hospital, Hialeah, USA; 2 Medicine, Nova Southeastern University Dr. Kiran C. Patel College of Osteopathic Medicine, Fort Lauderdale, USA; 3 Family Medicine, Nova Southeastern University Dr. Kiran C. Patel College of Osteopathic Medicine, Fort Lauderdale, USA

**Keywords:** cerebrovascular disease, encephaloduroarteriosynangiosis (edas), exchange transfusion, moyamoya syndrome, sickle cell disease, therapeutic phlebotomy

## Abstract

Moyamoya syndrome is a rare, progressive cerebrovascular disorder characterized by stenosis or occlusion of the terminal internal carotid arteries with the formation of fragile collateral vessels. While classically described in pediatric patients and idiopathic Moyamoya disease, secondary Moyamoya syndrome has been reported in association with sickle cell disease (SCD). Adult presentations remain uncommon and are frequently underrecognized. We report a unique case of a 36-year-old man with sickle cell anemia who developed acute neurological deterioration following hematologic optimization with packed red blood cell transfusion and therapeutic phlebotomy during a vaso-occlusive crisis. Cerebral angiography revealed complete occlusion of the left internal carotid artery at its terminus, consistent with Moyamoya syndrome. The patient subsequently underwent surgical revascularization with encephaloduroarteriosynangiosis with subsequent neurological stabilization. This case illustrates how advanced but previously compensated large-vessel cerebrovascular disease in adults with SCD may become clinically manifest during physiologic perturbation, underscoring the importance of early vascular imaging and multidisciplinary management when neurologic symptoms evolve during hematologic therapy.

## Introduction

Sickle cell disease (SCD) is associated with a broad spectrum of cerebrovascular complications resulting from chronic hemolysis, endothelial dysfunction, inflammation, and a prothrombotic state. Stroke is a well-established complication in pediatric SCD populations, with an estimated incidence of approximately 11% by age 20 in untreated children, prompting routine surveillance with transcranial Doppler ultrasonography and prophylactic transfusion strategies [1]. In contrast, cerebrovascular risk in adults with SCD is less clearly defined, and structured screening protocols are not routinely implemented, despite growing evidence of substantial subclinical disease burden.

Moyamoya vasculopathy is characterized angiographically by progressive stenosis or occlusion of the terminal internal carotid arteries and their proximal branches, accompanied by the formation of fragile collateral vessels producing the classic “puff of smoke” appearance [2]. Moyamoya disease refers to an idiopathic, typically bilateral process, whereas Moyamoya syndrome describes a similar angiographic pattern occurring in association with an underlying condition, including SCD, Down syndrome, neurofibromatosis, cranial irradiation, and autoimmune disorders [3]. This distinction is particularly relevant in cases with unilateral involvement, as seen in secondary forms.

In SCD, chronic intravascular hemolysis leads to nitric oxide depletion, oxidative stress, and endothelial activation. Recurrent vaso-occlusive episodes further promote smooth muscle proliferation, intimal hyperplasia, and progressive large-vessel stenosis, a pathophysiologic process distinct from acute microvascular occlusion [3,4]. Over time, these changes predispose patients to intracranial arteriopathy involving the internal carotid artery terminus and its branches.

Although Moyamoya syndrome is well documented in pediatric SCD cohorts, adult presentations remain relatively rare in the literature. Retrospective analyses suggest that cerebrovascular abnormalities, including silent cerebral infarcts, intracranial stenosis, and aneurysms, are prevalent in adults with SCD, yet often remain clinically occult [5]. The absence of routine adult screening may allow advanced vasculopathy to remain compensated until a physiologic stressor alters cerebral hemodynamics.

Hematologic interventions such as transfusion, exchange therapy carried out in case of cardiopulmonary bypass in SCD, and phlebotomy are cornerstone strategies in SCD management [6]. Rapid shifts in hemoglobin concentration, blood viscosity, and oxygen delivery may influence cerebral perfusion, particularly in patients with pre-existing large-vessel stenosis. In pediatric cardiac surgery for SCD, perioperative factors such as hypoxia, hypothermia, acidosis, and low-flow states are recognized contributors to vascular occlusion and thrombosis, highlighting the sensitivity of this population to hemodynamic perturbations [7].

We present a case of adult-onset Moyamoya syndrome unmasked during hematologic optimization in a patient with sickle cell anemia, emphasizing the diagnostic challenges, physiologic considerations, and importance of early neurovascular evaluation in adult SCD patients with evolving neurologic symptoms.

## Case presentation

A 36-year-old man with a known history of sickle cell anemia presented to the emergency department with diffuse joint pain, chest discomfort, and generalized body aches consistent with a vaso-occlusive crisis. He denied focal neurologic deficits at the time of presentation.

Initial laboratory evaluation demonstrated normocytic anemia with biochemical evidence of active hemolysis, including elevated lactate dehydrogenase, indirect hyperbilirubinemia, and reduced haptoglobin (Table 1). Iron studies revealed markedly elevated ferritin and transferrin saturation, consistent with chronic transfusional exposure and inflammation.

**Table 1:** Lab Values gm/dL, grams per deciliter; fL, femtoliter; mcg/dL, micrograms per deciliter; mg/dL, milligrams per deciliter; ng/mL, nanograms per milliliter; U/L, units per liter; TIBC, total iron binding capacity; AST, aspartate aminotransferase; ALT, alanine aminotransferase.

Test (Unit)	Observed Value	Normal Range
Hemoglobin (g/dL)	7.7	14.0-18.0
Mean Corpuscular Volume (fL)	86	80-94
Iron (mcg/dL)	178	49.0-181.0
TIBC (mcg/dL)	196	261-462
Saturation (%)	91	20-55
Transferrin (mg/dL)	336.7	206.0-381.0
Ferritin (ng/mL)	4090	17.9-464.0
AST (U/L)	84	17-59
ALT (U/L)	56	21-72
Alkaline Phosphatase (U/L)	134	38-126
Lactate Dehydrogenase (U/L)	600	120-246
Total Bilirubin (mcg/dL)	10.8	0.20-1.30

The patient, whose blood type was O positive, received one unit of packed red blood cells followed by therapeutic phlebotomy of approximately 600 mL as part of hematologic optimization. Pre-intervention hemoglobin was 7.7 g/dL, with post-intervention hemoglobin rising to 9.4 g/dL prior to phlebotomy and stabilizing thereafter. Post-intervention hemoglobin electrophoresis demonstrated hemoglobin A of 71.3% and hemoglobin S of 25.3%, achieving exchange-based therapeutic targets [8] (Table 2). Comparison is limited by the absence of a baseline hemoglobin electrophoresis before the intervention.

**Table 2:** Post-Intervention Hemoglobin Electrophoresis % = percent

Test (Unit)	Observed Value	Normal Range
Hemoglobin A (%)	71.3	96.4-98.8
Hemoglobin A2 (%)	2.6	1.8-3.2
Hemoglobin F (%)	0.8	0.0-2.0
Hemoglobin S (%)	25.3	0.0-0.0
Hemoglobin Solubility	Positive	Negative

Shortly after completion of hematologic interventions, the patient developed progressive alteration in mental status characterized by confusion, decreased responsiveness, and impaired attention. Following the clinical deterioration, the patient was closely monitored by neurology. The neurological assessment revealed a distinct clinical profile: the patient was aphonic, with no vocal sounds, yet was able to follow commands, read, and communicate via text. Cranial nerve examination showed full visual fields by confrontation, extraocular movements were intact (EOMI), and pupils were equal, round, and reactive to light and accommodation (PERRLA). Motor examination demonstrated mild right-sided hemiparesis, with the patient able to maintain the right upper and lower limbs against gravity. Notably, the patient exhibited significant weakness in the left lower extremity, characterized by an obvious drift and inability to hold the leg against gravity. Sensory and cerebellar examinations were otherwise unremarkable, and there were no pathological reflexes (no Babinski sign) [9]. Neurological status was monitored using serial Glasgow Coma Scale (GCS) assessments, pupillary examination, and continuous cardiorespiratory monitoring, consistent with established principles of postoperative neurosurgical care [9].

**Figure 1:**
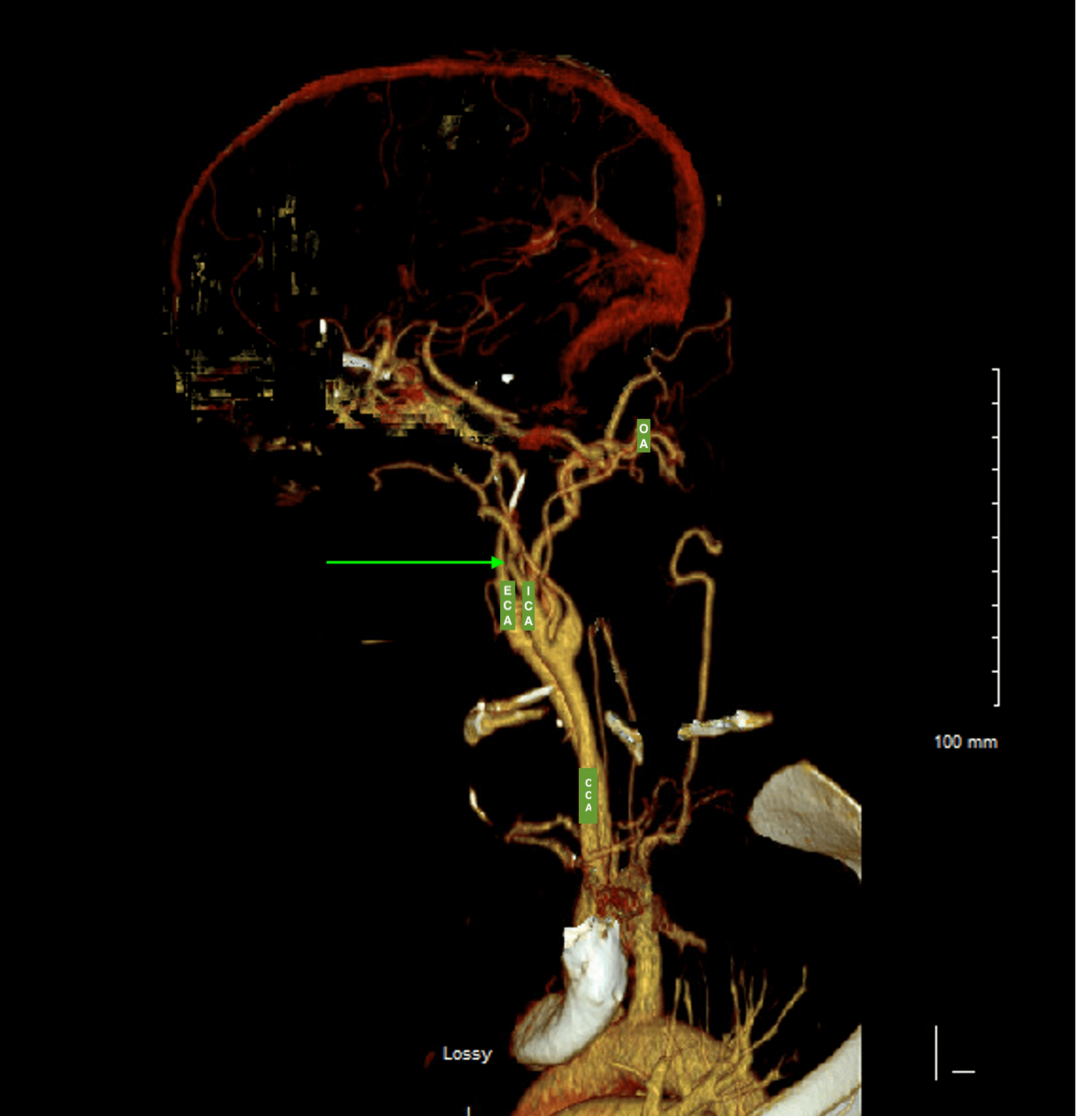
Computed tomography angiography of the head and neck with intravenous contrast Green arrow indicates left internal carotid artery (ICA) occlusion. Labels include CCA (common carotid artery), ECA (external carotid artery), ICA (internal carotid artery), and OA (occipital artery).

Cerebral angiography revealed complete occlusion of the left internal carotid artery near its terminus with extensive collateral vessel formation consistent with Moyamoya syndrome (Figure 1). These findings suggested chronic vascular remodeling rather than an acute embolic event.

**Figure 2:**
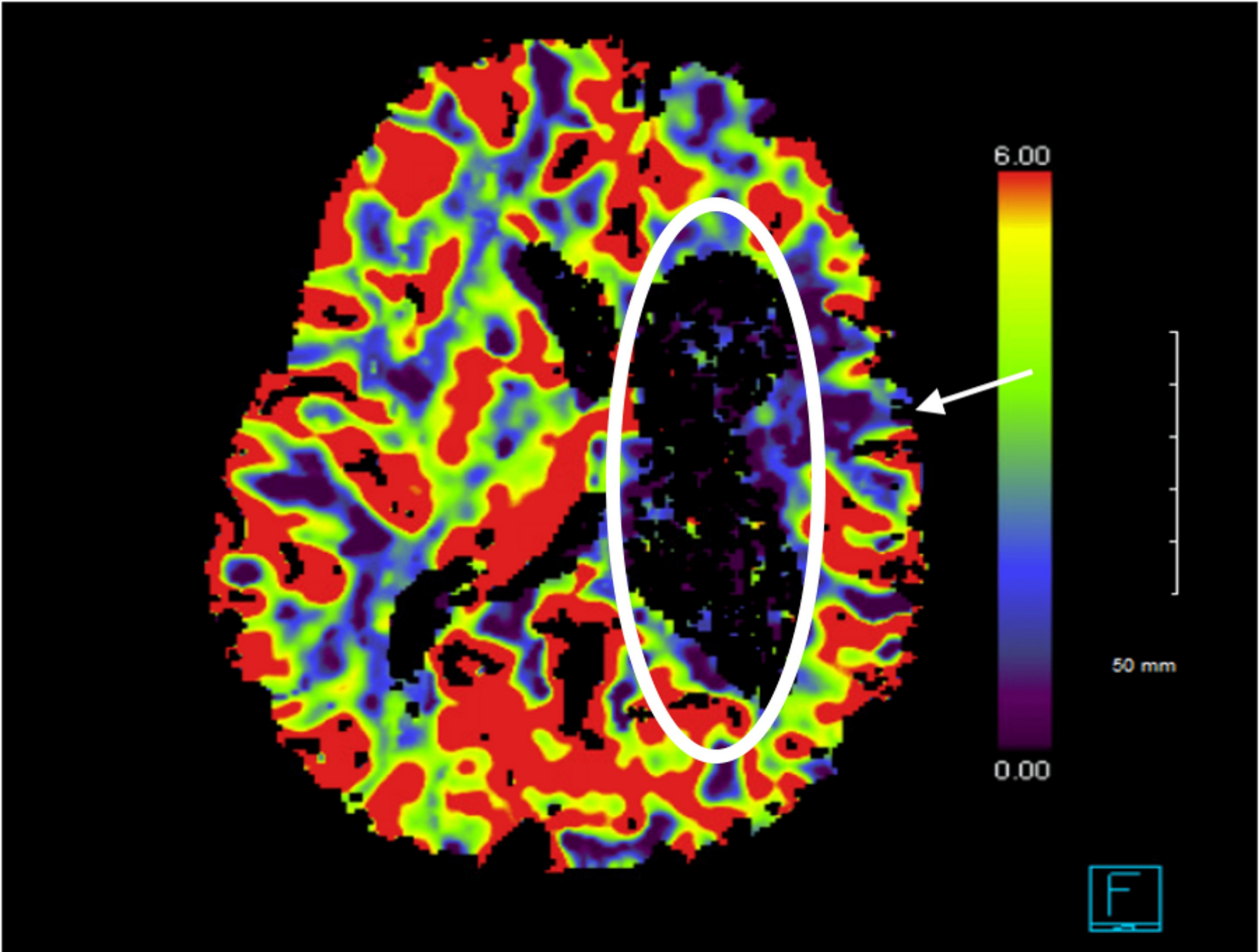
Computed tomography cerebral perfusion imaging with intravenous contrast White arrow is pointing to areas of decreased blood flow and volume identified within the left posterior temporal and parietal cortical subcortical region, and the white oval is encircling the dilated left lateral ventricle.

Cerebral perfusion imaging revealed areas of decreased blood flow and volume identified within the left posterior temporal and parietal cortical subcortical region, and a dilated left lateral ventricle (Figure 2). These findings supported the lack of perfusion evident on cerebral angiography.

The patient was evaluated by neurosurgery and subsequently underwent surgical revascularization via encephaloduroarteriosynangiosis (EDAS). EDAS is an indirect revascularization technique designed to augment cerebral perfusion by promoting neoangiogenesis. The procedure involves the transposition of a donor scalp artery, typically the superficial temporal artery (STA), onto the surface of the ischemic brain. Unlike direct bypass (superficial temporal artery-middle cerebral artery bypass), EDAS does not require a microvascular anastomosis. Instead, the STA is meticulously dissected, maintaining its proximal and distal continuity to preserve flow. A craniotomy is performed, and the dura mater is opened to expose the underlying cortex. The living donor vessel is then laid directly onto the arachnoid surface and secured to the dural edges. Over a period of several months, the chronically ischemic environment of the brain stimulates the formation of new collateral vessels from the donor artery [10].

Anesthesia and pain management

For the surgical revascularization procedure, anesthesia was induced using intravenous propofol and fentanyl, followed by neuromuscular blockade using rocuronium to facilitate endotracheal intubation. Maintenance anesthesia consisted of a balanced technique using inhalational anesthetics and opioid analgesia. Intraoperative findings confirmed successful collateralization via superficial temporal artery branches. Postoperatively, neurological status was monitored using serial GCS assessments, pupillary examination, and continuous cardiorespiratory monitoring, consistent with established principles of postoperative neurosurgical care [9]. The patient’s neurologic status stabilized with gradual improvement in mental function and no further progression of deficits.

Postoperative analgesia was managed using a multimodal pain control strategy designed to provide adequate analgesia while preserving neurologic assessment. The patient received intravenous acetaminophen (1,000 mg every 6 hours) as a scheduled non-opioid analgesic foundation. For breakthrough pain, intravenous hydromorphone (0.2-0.5 mg every three to four hours as needed) was administered in carefully titrated doses to avoid oversedation. Following stabilization and extubation, the patient was transitioned to oral oxycodone (5 mg every four to six hours as needed) for continued pain control. Adjunctive therapy included low-dose gabapentin (100-300 mg nightly) for neuropathic discomfort and to reduce opioid requirements. Nonsteroidal anti-inflammatory drugs (NSAIDs) were avoided in the immediate postoperative period due to concern for bleeding risk following intracranial surgery. Pain control was closely coordinated with serial neurologic examinations to ensure that analgesic therapy did not obscure changes in mental status or focal deficits.

## Discussion

This case highlights a rare adult presentation of Moyamoya syndrome (MMS) in a patient with SCD undergoing active hematologic therapy. While MMS is a well-documented cause of pediatric stroke in SCD, adult prevalence data suggests a significant, often subclinical, cerebrovascular burden. Cohort studies indicate that intracranial stenosis and silent cerebral infarcts (SCI) persist into adulthood, with some studies estimating SCI prevalence as high as 50% in adults with SS genotypes [11]. This suggests that advanced vasculopathy may remain hemodynamically compensated until unmasked by acute physiologic stressors.

The pathophysiology of MMS in SCD involves a distinct progression from microvascular vaso-occlusive injury to large-vessel arteriopathy. Chronic hemolysis leads to free hemoglobin-mediated nitric oxide depletion and oxidative stress, which triggers endothelial dysfunction and smooth muscle cell proliferation [4]. In the internal carotid terminus, this manifests as intimal hyperplasia and progressive stenosis. While our patient presented with unilateral disease, it is essential to note that MMS in SCD can be asymmetric [12]; however, the potential for future contralateral involvement necessitates rigorous longitudinal monitoring and clarifies the diagnostic distinction from idiopathic Moyamoya disease.

The patient’s neurologic deterioration following transfusion and therapeutic phlebotomy suggests a complex hemodynamic shift. While we hypothesize that rapid changes in blood viscosity and oxygen-carrying capacity may have unmasked a state of compensated hypoperfusion, we acknowledge the limitation of missing serial hematocrit and volume status markers to definitively establish causality. However, literature on cerebral autoregulatory dysfunction suggests that in the setting of fixed, high-grade stenosis, the brain’s ability to maintain constant blood flow is severely impaired [13]. Rapid hematocrit correction may paradoxically alter rheology or cerebral metabolic demand, potentially leading to "misery perfusion" or focal ischemia in watershed territories already reliant on fragile collateral networks.

Regarding management, surgical revascularization remains the definitive treatment for symptomatic MMS. While indirect methods like EDAS are standard in pediatrics, the choice between direct (STA-MCA bypass) and indirect bypass in adults remains a subject of debate. The superficial temporal artery to middle cerebral artery (STA-MCA) bypass can be used to augment flow to the distal MCA vascular territory [10]. Adult outcomes for EDAS show reduced stroke recurrence, though perioperative management requires meticulous blood pressure control and aggressive transfusion strategies to maintain hepatitis B surface antibody (HbS) levels below 30% and prevent graft failure [10].

## Conclusions

This case illustrates a rare and clinically significant presentation of Moyamoya syndrome in an adult patient with SCD. The temporal association between hematologic optimization and neurologic deterioration suggests a potential hemodynamic unmasking of advanced, previously subclinical large-vessel vasculopathy rather than a direct complication of therapy. While the absence of serial quantitative perfusion metrics precludes a definitive causal link between viscosity shifts and acute ischemia, this case highlights how rapid physiologic perturbations may reveal a state of exhausted cerebrovascular reserve in the setting of chronic steno-occlusive disease.

Ultimately, the recognition of Moyamoya syndrome in the adult SCD population remains essential. This case demonstrates that even in patients undergoing appropriate hematologic management, a high index of clinical suspicion for underlying intracranial vasculopathy is required when neurologic symptoms emerge. Early diagnostic imaging and multidisciplinary coordination for surgical revascularization are critical interventions to stabilize cerebral blood flow and prevent catastrophic cerebrovascular events in this high-risk demographic.
